# Analysis of Mathematical Modelling on Potentiometric Biosensors

**DOI:** 10.1155/2014/582675

**Published:** 2014-05-07

**Authors:** N. Mehala, L. Rajendran

**Affiliations:** ^1^Department of Mathematics, K.L.N. College of Engineering, Sivagangai, Tamil Nadu, India; ^2^Department of Mathematics, The Madura College, Madurai, Tamil Nadu 625 011, India

## Abstract

A mathematical model of potentiometric enzyme electrodes for a nonsteady condition has been developed. The model is based on the system of two coupled nonlinear time-dependent reaction diffusion equations for Michaelis-Menten formalism that describes the concentrations of substrate and product within the enzymatic layer. Analytical expressions for the concentration of substrate and product and the corresponding flux response have been derived for all values of parameters using the new homotopy perturbation method. Furthermore, the complex inversion formula is employed in this work to solve the boundary value problem. The analytical solutions obtained allow a full description of the response curves for only two kinetic parameters (unsaturation/saturation parameter and reaction/diffusion parameter). Theoretical descriptions are given for the two limiting cases (zero and first order kinetics) and relatively simple approaches for general cases are presented. All the analytical results are compared with simulation results using Scilab/Matlab program. The numerical results agree with the appropriate theories.

## 1. Introduction

Electrochemical biosensors are used as detectors in several commercial analyzers for the accurate and rapid determination of various metabolites such as urea, glucose, lactate, and creatinine [1–5]. These biosensors are fabricated by immobilizing appropriate bioreagents (i.e., enzymes) in a layer adjacent to the sensing surface of the basic electrochemical transducers. The enzyme layer catalyzes the conversion of metabolite molecules, ultimately consuming or producing an electrochemically detectable species. Thus, the analytical performance of these biosensor systems is largely dependent upon the properties of the immobilized enzyme layer incorporated.

A biosensor is an analytical device comprising of a biological element capable to recognize an analyte, coupled with a transducer which generates a signal proportional to the concentration of the analyte and it combines the selectivity and specificity of an immobilized biologically active compound with a signal transducer [6–8]. The analytical application of biosensors has become a focus of interest and a subject of rapid progress [9–11]. The theory of enzyme-based potentiometric sensors is being treated in a series of pioneering contributions. Explicit solutions were derived by Blaedel et al. [12] for the steady state response of such electrodes which apply either to very high or to sufficiently low substrate concentrations. Carr followed the same results from a Fourier analysis as limiting cases for long periods [13, 14].

A relatively simple approach was presented by Morf and he obtained an explicit result for the electrode response that applies to the whole range of substrate concentration [15, 16]. The response behaviors of potentiometric enzyme electrodes as well as the product release from enzyme reactors were treated for the steady-state case in paper [17] and for the nonsteady state in paper [18] by the same author. Numerical simulations were developed for modeling the reaction and diffusion processes that arise in the functional enzyme membranes of such systems. These simulations represent a kind of virtual experiments and they allowed it to get insight into the concentration profiles and fluxes of substrate and product species and to analyze the final response characteristics of enzyme-based sensors and reactors [11, 19].

To our knowledge, no general analytical expressions of the concentrations of the substrate, product, and current have been reported for all values of parameters [13]. The purpose of this communication is to derive the concentrations of the substrate, product, and current for all values of reaction parameters using homotopy perturbation method. The theoretical treatments make use of the homotopy perturbation method and lead to relationships for all decisive quantities as a function of time. The theoretical results agree with simulated data and offer the basis for reliable predictions of response time ranges for enzyme electrodes and enzyme reactors.

## 2. Mathematical Formation of the Problem

In this paper, we consider the analytical system based on an enzyme-containing bulk membrane of thickness *d* that contains a uniform total concentration of the enzyme *E* which is contacted on one side with an aqueous solution of the substrate *S*. The substrate molecules diffuse into the membrane phase where they react in accordance with Michaelis-Menten type enzyme catalyzed reaction [20, 21] to yield an electroactive product *P*. Consider

(1) $$\begin{array}{c} E+S\overset{k_{1}}{\underset{k_{2}}{⇄}}ES\overset{k_{3}}{⟶}E+\upsilon P, \end{array}$$

where

(2) KM=  k2+k  3  k1  ,    

where *ES* is the intermediate enzyme-substrate complex, *υ* is the number of product species obtained per substrate molecule, *k*_1_, *k*_2_, and *k*_3_ are the rate constants of the respective partial reactions, and *K*_*M*_ is the Michaelis constant defined in (2). The influences of reaction and diffusion processes for the species *S* and *P* in the enzyme membrane are described by the following nonlinear governing equations:

(3) $$\begin{array}{c} \frac{\partial {\left[S\right]}_{\text{em}}}{\partial t}=D_{s}\frac{\partial^{2}{\left[S\right]}_{\text{em}}}{\partial x^{2}}-k_{3}{\left[E\right]}_{\text{tot}}\frac{{\left[S\right]}_{\text{em}}}{{\left[S\right]}_{\text{em}}+K_{M}}\\ \frac{\partial {\left[P\right]}_{\text{em}}}{\partial t}=D_{p}\frac{\partial^{2}{\left[P\right]}_{\text{em}}}{\partial x^{2}}+\upsilon k_{3}{\left[E\right]}_{\text{tot}}\frac{{\left[S\right]}_{\text{em}}}{{\left[S\right]}_{\text{em}}+K_{M}}, \end{array}$$

where [*S*]_em_ and [*P*]_em_ are the concentrations of the species in the enzyme membrane, *D*_*s*_ and *D*_*p*_ are the corresponding diffusion coefficients, [*E*]_tot_ is the total concentration of free enzymes and enzyme-substrate complexes that is assumed to be constant within the membrane including surface zones, *υ* is the number of product species obtained per substrate molecule, and *k*_3_ is the rate constant for the irreversible step of product formation [17, 18]. Now, (3) are solved by assuming the zero fluxes at *x* = 0 and of equilibrium distribution at *x* = *d* [17, 18]. The initial state is given by zero concentrations of substrate and product species throughout [17]. Consider

(4) $$\begin{array}{c} \frac{\partial {\left[S\right]}_{\text{em}}}{\partial x}=0,\;\frac{\partial {\left[P\right]}_{\text{em}}}{\partial x}=0\;\text{when}\;\;x=0\\ {\left[S\right]}_{\text{em}}=k_{s}{\left[S\right]}_{\text{aq},}\;{\left[P\right]}_{\text{em}}=k_{p}{\left[P\right]}_{\text{aq}\;}\;\text{when}\;\;x=d\\ {\left[S\right]}_{\text{em}}=0,\;{\left[P\right]}_{\text{em}}=0\;\text{when}\;\;t=0.\; \end{array}$$

For enzyme reactors, the outward flux of the product species at *x* = *d* is described by

(5) $$\begin{array}{c} J_{p}={-D_{p}\frac{\partial {\left[P\right]}_{\text{em}}}{\partial x}|}_{x=d}\;\;, \end{array}$$

where *D*_*p*_ is the diffusion coefficient of the product.

## 3. Dimensionless Form of the Problem

To compare the analytical results with the simulation results, we make the above nonlinear partial differential equations (3) in dimensionless form by defining the following parameters:

(6) $$\begin{array}{c} u=\frac{{\left[S\right]}_{\text{em}}}{k_{s}{\left[S\right]}_{\text{aq}}},\;\;v=\frac{{\left[P\right]}_{\text{em}}}{k_{s}{\left[S\right]}_{\text{aq}}},\;\;X=\frac{x}{d},\;\\ \alpha =\frac{k_{s}{\left[S\right]}_{\text{aq}}}{k_{M}},\;\;\;\beta =\frac{k_{p}{\left[P\right]}_{\text{aq}}}{k_{M}}\\ \gamma_{s}=\sqrt{\frac{kd^{2}}{D_{s}}},\;\;\;k=\frac{k_{3}{\left[E\right]}_{\text{tot}}}{K_{M}},\;\;\;\tau =\frac{Dt}{d^{2}}. \end{array}$$

Here, we assume that *D*_*s*_ = *D*_*p*_ = *D*.

Equations (3) reduce to the following dimensionless form:

(7) $$\begin{array}{c} \;\;\frac{\partial u}{\partial \tau }=\frac{\partial^{2}u}{\partial X^{2}}-\frac{\gamma_{s}^{2}u}{\alpha u+1}\\ \frac{\partial v}{\partial \tau }=\frac{\partial^{2}v}{\partial X^{2}}+\frac{\upsilon \gamma_{s}^{2}u}{\alpha u+1}, \end{array}$$

where *u* and *v* represents the dimensionless concentration of substrate and product, *α* and *β* are saturation parameters, and *γ*_*s*_ is the reaction diffusion parameter (Thiele modulus). Now, the boundary conditions may be presented as follows [17, 18]:

(8) $$\begin{array}{c} \frac{\partial u}{\partial X}=0,\;\;\frac{\partial v}{\partial X}=0\;\text{when}\;\;\;X=0 \end{array}$$

(9) $$\begin{array}{c} u=1,\;v=\frac{\beta }{\alpha }\;\text{when}\;\;X=1 \end{array}$$

(10) $$\begin{array}{c} u=0,\;v=0\;\text{when}\;\;\tau =0. \end{array}$$

The normalized flux becomes

(11) $$\begin{array}{c} \psi =\frac{J_{p}d}{Dk_{s}{\left[S\right]}_{\text{aq}}}=-{\left|\frac{\partial v}{\partial X}\right|}_{X=1}. \end{array}$$

## 4. Analytical Expressions for the Concentrations and Current for All Values of Parameters

By using Laplace transform technique and new Homotopy perturbation method (Appendix A), we can obtain the concentrations of substrate and product as follows:

(12) u(x,τ)=cosh⁡(aX)cosh⁡(a) −∑m=0∞(π(−1)m(2m+1)fm)e−(fmτ)cos⁡(2m+1)πX2

(13) v(x,τ)=(βα+υ)(1−4π∑m=0∞[((−1)m2m+1)e−(π2(2m+1)2/4)τkkkkkkkkkkkkkkkkkkk×1−4π∑m=0∞[((−1)m2m+1)e−(π2(2m+1)2/4)τcos⁡(2m+1)πX2((−1)m2m+1)]) −υu(x,τ),

where

(14) $$\begin{array}{c} f_{m}=\frac{\pi^{2}{\left(2m+1\right)}^{2}+4a}{4},\;a=\frac{\gamma_{s}^{2}}{1+\alpha }. \end{array}$$

The analytical expression for the dimensionless current is given by

(15) ψ=(βα+υ)[∑m=0∞2(−1)me(−π2(2m+1)2/4)τ] −υ[atanha+∑m=0∞2π2(2m+1)2e−(fm)τπ2(2m+1)2+4a],

where *f*_*m*_ and *a* are defined as in (14).

## 5. Analytical Expressions for the Concentrations and Current for Unsaturated (First Order) Kinetics

Now, we consider the limiting case where the substrate concentration is relatively low. In this case, *αu* ≤ 1  (i.e., [*S*]_em  _ ≤ *K*_*M*_). Then, (7) will be reduced to the following dimensionless form:

(16) $$\begin{array}{c} \frac{\partial u}{\partial \tau }=\frac{\partial^{2}u}{\partial X^{2}}-\gamma_{s}^{2}u\\ \frac{\partial v}{\partial \tau }=\frac{\partial^{2}v}{\partial X^{2}}+\upsilon \gamma_{s}^{2}u. \end{array}$$

The dimensionless form of concentrations obtained by Morf et al. [17] using method of expressions in partial fractions is as follows:

(17) u(x,τ)=  cosh⁡(γsX)cosh⁡(γs) −π∑n=0∞(−1)n(2n+1fn)cos⁡(2n+1)πX2e−fnτ,

where

(18) $$\begin{array}{c} f_{n}=\frac{{\left(2n+1\right)}^{2}\pi^{2}}{4}+\gamma_{s} \end{array}$$

(19) v(x,τ)=(υ+βα)[1−4π∑n=0∞(−1)n2n+1kkkkkkkkkk×cos⁡(2n+1)πX2e−((2n+1)2π2/4)τ1−4π∑n=0∞(−1)n2n+1] −υ[u(x,τ)].

The analytical expression for the dimensionless current is given by

(20) ψ(τ)=υ(γstanhγs+π22∑n=0∞(2n+1)2fne−fnτ) −(υ+βα)[2∑n=0∞e−((2n+1)2π2τ/4)].

## 6. Analytical Solutions for the Concentrations and the Current for Saturated (Zero Order) Kinetics

Next, we consider the limiting case where the substrate concentration is relatively high. In this case, *αu* ≥ 1  ([*S*]_em  _ ≥ *K*_*M*_). Equations (7) will be reduced to the following form:

(21) $$\begin{array}{c} \frac{\partial u}{\partial \tau }=\frac{\partial^{2}u}{\partial X^{2}}-\frac{\gamma_{s}^{2}}{\alpha }\\ \frac{\partial v}{\partial \tau }=\frac{\partial^{2}v}{\partial X^{2}}+\frac{\upsilon \gamma_{s}^{2}}{\alpha }. \end{array}$$

The analytical expressions for the concentrations of substrate and product are as follows [22]:

(22) u(X,τ)=1+γs2(X2−1)2α +∑n=1∞(−1)n(16γs2π3α(2n−1)3−4π(2n−1)) ×cos⁡⁡((n−1)πX2)[e−((n−1)/2)2π2τ]v(X,τ)=1−υγs2(X2−1)2α +∑n=1∞(−1)n(16γs2π3α(2n−1)3−4π(2n−1)) ×cos⁡⁡((n−1)πX2)[e−((n−1)/2)2π2τ].

The analytical expression for the dimensionless current is given by

(23) ψ(τ)=υγs22α +∑n=1∞2(−1)n(n−1)(4γs2π2α(2n−1)3−1(2n−1)) ×sin⁡((n−12)π)[e−((n−1/2)2π2)τ].

## 7. Numerical Simulation

**Figure 1:**
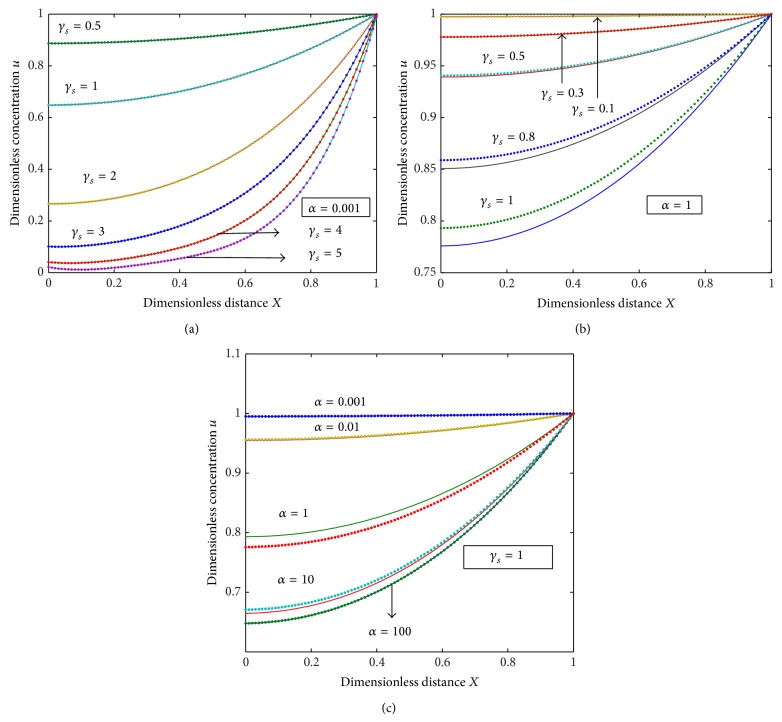
Plot of dimensionless nonsteady concentration profiles of the substrate *u* versus dimensionless distance *X* for various values of the parameters *γ*_*s*_ and *α*, when *τ* = 100. Solid lines represent the numerical simulation and the dotted lines represent the analytical solution (12).

**Figure 2:**
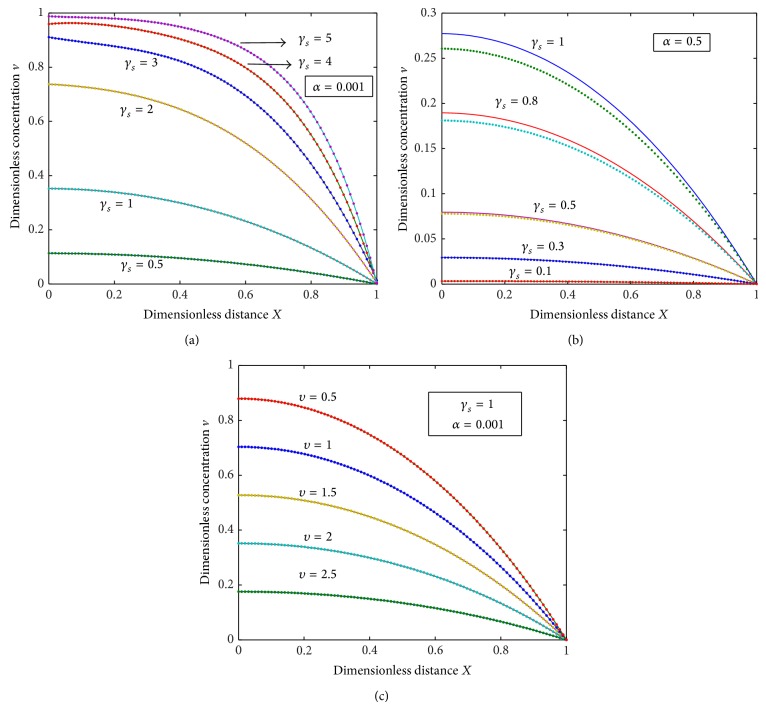
Plot of dimensionless nonsteady concentration profiles of the product *v* versus dimensionless distance *X* for various values of the parameters *γ*_*s*_, *α*, and *υ*, when *τ* = 100. Solid lines represent the numerical simulation and the dotted lines represent the analytical solution (13).

**Figure 3:**
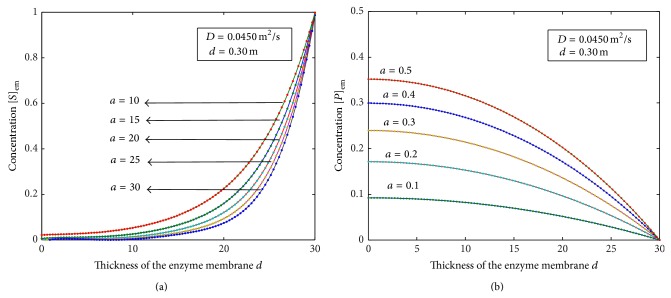
Plot of nonsteady concentration profiles of the substrate and product versus thickness of the membrane *d* when *τ* = 1 and for various values of the parameters *a* and *D*. Solid lines represent the numerical simulation and the dotted lines represent the analytical solutions (6), (12), and (13).

The diffusion equations (7) for the corresponding boundary conditions (8), (9), and (10) are solved by numerical methods. The function pdex 4 in Matlab software, which is a function of solving the initial boundary value problems for partial differential equations, was used to solve these equations numerically (Appendix A). The numerical solutions are compared with our analytical results as shown in Figures 1, 2, 3, and 5 and this comparison gives a satisfactory agreement for some possible values of the reaction diffusion parameters.

## 8. Results and Discussions

Equations (12) and (13) are the new analytical expressions of concentrations of substrate and product for all values of parameters *γ*_*s*_ and *α*. The previously reported analytical results ((17) and (19)) are in terms of the parameter *γ*_*s*_ only.

Figure 1 shows the time-dependent evolution of normalized concentration profiles for the substrate *u* in the enzyme membrane of a potentiometric sensor. Figures 1(a)–1(c) show dimensionless concentration *u* versus the dimensionless distance *X*. The reaction diffusion parameter *γ*_*s*_ is an indicator of the competition between the reaction and diffusion. When *γ*_*s*_ is small, the kinetics dominate and the uptake of the substrate are kinetically controlled. From Figure 1(a), it is evident that the value of the substrate concentration *u* decreases when the reaction diffusion parameter *γ*_*s*_ increases for different values of *α*.  Figure 1(b) illustrates that, when *γ*_*s*_ increases, the concentration of the substrate *u* decreases even though the value of *α* is increased. It is obvious from Figure 1(c) that when *α* increases the concentration *u* decreases and if *α* is very small, the concentration of the substrate is uniform and the curve becomes a straight line. Recently, Sivasankari and Rajendran [23] discussed the same mathematical model of potentiometric biosensors for the steady state and according to them when the diffusion parameter *γ*_*s*_ is very small, the diffusion of the substrate concentration will be uniform and the curve becomes straight line. This reveals that the parameter time *t* has greater impact on diffusion.

The normalized concentration of the product *v* for various values of *α* and *υ* is plotted in Figures 2(a)–2(c). From the figures we can conclude that the normalized product *v* increases with the decrease in the value of *υ* and increases with the increase in the value of *α*. Figures 3(a) and 3(b) show the evolution of concentration profiles of the substrate [*S*]_em_ and the product [*P*]_em_ for the experimental values of the diffusion coefficient *D*, for the thickness of the membrane *d*, and for various values of *a*. From the figures, it is inferred that the concentration of the substrate increases slowly when *d* ≤ 20 and increases sharply when 20 ≤ *d* ≤ 30. The values of the concentrations of the substrate do not differ significantly for all values of *a* whereas the values of the concentration of the product differ significantly for some values of *a*.

**Figure 4:**
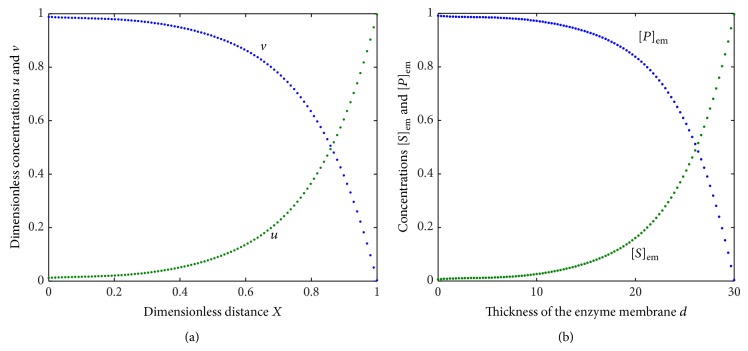
(a) Plot of dimensionless concentrations *u* and *v* versus dimensionless distance *X* when the dimensionless parameter *γ*_*s*_ = 5, *υ* = 1, and *a* = 0.001 using (12) and (13). (b) Plot of concentrations [*S*]_em_ and [*P*]_em_ versus the thickness of the membrane *d* for the experimental values of the parameters  *D* = 0.045 m^2^/s, *υ* = 1, and *a* = 15 using (6), (12), and (13).

Figure 4(a) indicates the normalized concentration of the substrate and product for the approximate analytical values of the parameters which reveals that, at time *t* = 0, the membrane surface at *X* = 30 is brought in contact with a substrate sample. The substrate molecules then start to diffuse into the enzyme layer whereas Figure 4(b) represents the concentration of the substrate and product for the experimental values of the parameters involved in the solutions of the nonlinear differential equations (3). The figure infers that the catalytic reaction generates an increasing concentration of product species towards the side of the indicator electrode at *X* = 0. Also, from the figure, it is confirmed that *u* + *v* = 1 or [*S*]_em_ + [*P*]_em_ = 1, for all values of time and also for all values of the parameters involved.

**Figure 5:**
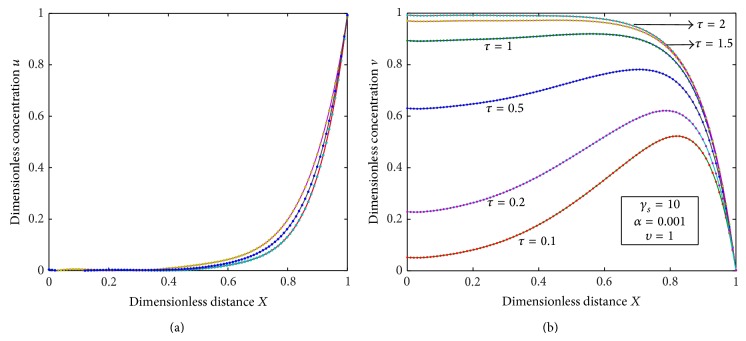
(a) Plot of dimensionless concentrations *u* versus dimensionless distance *X* for various values of *τ*, *γ*_*s*_, and *α* from bottom to top using (12). (b) Plot of dimensionless concentrations *v* versus dimensionless distance *X* for various values of *τ*, *γ*_*s*_, *α*, and *υ* using (13).

**Figure 6:**
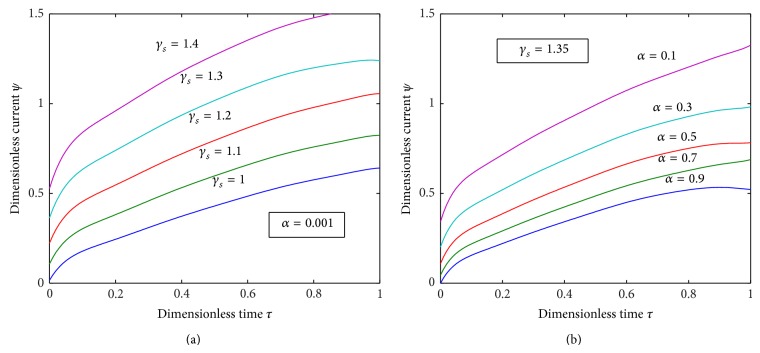
Plot of dimensionless current *ψ* versus dimensionless time *τ* for various values of the parameters *γ*_*s*_ and *α* using (15).

Figure 5(a) exhibits that the dimensionless concentration *u* increases as the dimensionless time *τ* increases and Figure 5(b) illustrates that the dimensionless concentration *v* also increases with the increase in the dimensionless time *τ*.  Figure 6(a) characterizes the dimensionless flux versus the dimensionless time for various values of the reaction diffusion parameter *γ*_*s*_ and for some *α* and the figure reveals that the value of the flux increases as *γ*_*s*_ increases, whereas Figure 6(b) exhibits that the value of flux increases as the value of the parameter *α* decreases.

**Figure 7:**
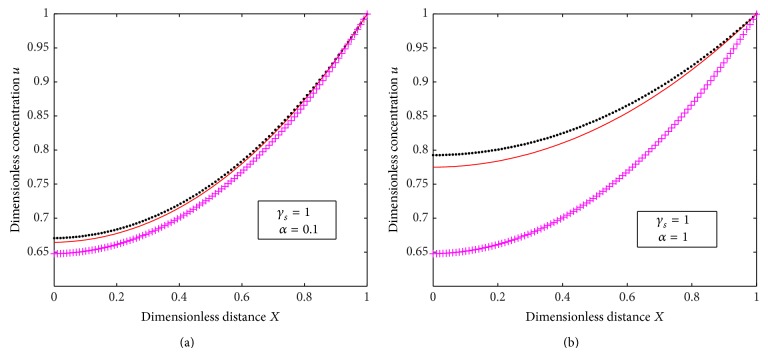
Plot of dimensionless concentration *u* versus dimensionless distance *X* for various values of *γ*_*s*_ and *α* where “orange straight line” represents numerical solution, “black dotted line” represents analytical solution of our work (12), and “purple dashed line” represents the analytical solution of Worf's work (17).

**Figure 8:**
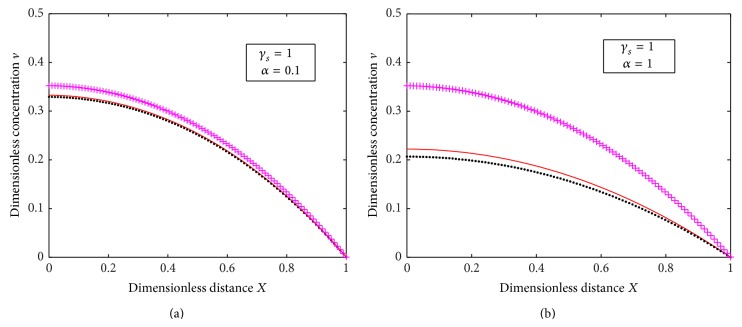
Plot of dimensionless concentration *v* versus dimensionless distance *X* for various values of *γ*_*s*_^  ^ and *α* where “orange straight line” represents numerical solution, “black dotted line” represents analytical solution of our work (13), and “purple dashed line” represents the analytical solution of Worf's work (19).

Our analytical results (12) and numerical results for substrate concentration are compared with previous analytical results (17) of Morf et al. [17] in Figure 7. It exhibits that when *α* is small there is a coincidence between both the results, whereas, when *α* is 1, there is a significant difference between both the results. The same observation for the product concentration is exhibited in Figure 8.

## 9. Conclusion

The theoretical analysis of behaviour of potentiometric biosensor was done. The coupled time-dependent nonsteady state nonlinear diffusion equations have been solved analytically and numerically. Moreover we have obtained analytical expressions for the substrate, product concentrations and steady state flux. A good agreement with numerical simulation data is noticed. These analytical results will be used in determining the kinetic characteristics of the biosensor. The theoretical model presented here can be used for the optimization of the design of the biosensor. Furthermore, based on the outcome of this work, there is a possibility of extending the procedure to find the approximate amounts of substrate and product concentrations and current for the reciprocal competitive inhibition process.

## Appendices

### A. Solution of (7) Using Complex Inversion Formula

In this appendix, we indicate how (12) and (13) are derived. Using new homotopy perturbation approach [24, 25], (7) can be written as

(A.1) $$\begin{array}{c} \frac{\partial u}{\partial \tau }=\frac{\partial^{2}u}{\partial X^{2}}-\frac{\gamma_{s}^{2}u}{\alpha u\left[X=1\right]+1}\\ \frac{\partial v}{\partial \tau }=\frac{\partial^{2}v}{\partial X^{2}}+\frac{\upsilon \gamma_{s}^{2}u}{\alpha u\left[X=1\right]+1}. \end{array}$$

By using (9) in (A.1), we get

(A.2) $$\begin{array}{c} \frac{\partial u}{\partial \tau }=\frac{\partial^{2}u}{\partial X^{2}}-au \end{array}$$

(A.3) $$\begin{array}{c} \frac{\partial v}{\partial \tau }=\frac{\partial^{2}v}{\partial X^{2}}+\upsilon au, \end{array}$$

where *a* is defined as in (14).

Now, by applying Laplace transform to (A.2) and to the conditions in (8), (9), and (10), we obtained the solution of (A.2) as

(A.4) $$\begin{array}{c} \bar{u}\;=\;\frac{\;\cosh\left(\sqrt{s+a}X\right)}{s\cosh\left(\sqrt{s+a}\right)}. \end{array}$$

In this appendix, we indicate how (A.4) may be inverted using the complex inversion formula. If $\bar{y}(s)$ represents the Laplace transform of a function *y*(*τ*), then, according to the complex inversion formula, we can state that

(A.5) y(τ)=12π∫c−i∞c+i∞exp⁡[sτ]y¯(s)ds=12πi∮cexp⁡⁡[sτ]y¯(s)ds,

where the integration in (A.5) is to be performed along a line *s* = *c* in the complex plane where *s* = *x* + *iy*. The real number *c* is chosen such that *s* = *c* lies to the right of all the singularities but is otherwise assumed to be arbitrary. In practice, the integral is evaluated by considering the contour integral presented on the right-hand side of (A.5), which is then evaluated using the so-called Bromwich contour. The contour integral is then evaluated using the residue theorem which states, for any analytic function *F*(*z*), that

(A.6) ∮cF(z)dz=2πi∑nRes[F(z)]z=z0,

where the residues are computed at the poles of the function *F*(*z*). Hence, from (A.6), we note that

(A.7) y(τ)=∑nRes[exp⁡[sτ]y¯(s)]s=s0.

From the theory of complex variables, we can show that the residue of a function *F*(*z*) at a simple pole at *z* = *a* is given by

(A.8) Res[F(z)]z=a=lim⁡z→a{(z−a)F(z)}.    

Hence, in order to invert (A.4), we need to evaluate

(A.9) Res[cosh⁡(s+a)Xscosh⁡(s+a)].

The poles are obtained from $s\cosh\sqrt{s+a}$ = 0. Hence, there is a simple pole at *s* = 0 and there are infinitely many poles given by the solution of the equation $\cosh\sqrt{s+a}$ = 0 and so

(A.10) $$\begin{array}{c} s_{n}=\frac{-\pi^{2}{\left(2n+1\right)}^{2}-4a}{4}\;\text{where}\;\;n=0,1,2,\ldots \end{array}$$

Hence, we note that

(A.11)  u(X,τ)=Res⌊scosh⁡(s+a)⌋s=0 +Res⌊scosh⁡(s+a)⌋s=sn.

The first residue in (A.11) is given by

(A.12) Res[scosh⁡(s+a)]s=0=lim⁡s→0[exp⁡(sτ)cosh⁡(s+a)Xscosh⁡(s+a)]=cosh⁡aXcosh⁡a.

The second residue in (A.11) is given by

(A.13) Res⌊scosh⁡(s+a)⌋s=sn =lim⁡s→sn[exp⁡⁡(sτ)cosh⁡(s+a)Xscosh⁡(s+a)] =lim⁡s→sn[exp⁡(sτ)cosh⁡(s+a)Xs(d/ds)cosh⁡(s+a)] =−∑m=0∞[(−1)mπ(2m+1)e−fnτcos⁡⁡((2m+1)πX/2)fm],

where *f*_*m*_ is defined as in (14). Here, we used cosh⁡(*iθ*) = cos⁡(*θ*) and sinh⁡(*iθ*) = *i*sin(*θ*). From (A.11), (A.12), and (A.13), we conclude that

(A.14) u(X,τ)=cosh⁡aXcosh⁡a −∑m=0∞[(−1)mπ(2m+1)e−fmτcos⁡((2m+1)πX/2)fm],

where *f*_*m*_ is defined as in (14). Similarly, we can solve (A.3) by using complex inversion formula.

### B. Matlab Program for the Summation of Series in (12)

The Matlab program for finding the numerical solution for (7) is as follows:

function pdex 4
m = 0;
x = linspace(0,1);
t = linspace(0,1);
sol = pdepe(m,@pdex4pde,@pdex4ic,@pdex4bc,x,t);
u1 = sol(:,:,1);
u2 = sol(:,:,2);
figure
plot(x,u1(end,:))
title(‘u1(x,t)')
xlabel(‘Distance x')
ylabel(‘u1(x,2)')
%–––––––––––––––––––––––––––––––––
figure
plot(x,u2(end,:))
title(‘u2(x,t)')
xlabel(‘Distance x')
ylabel(‘u2(x,2)')
%–––––––––––––––––––––––––––––––––
function [c,f,s] = pdex4pde(x,t,u,DuDx)
c = [1;1];
f = [1;1].∗DuDx;
r = 10;
a = 0.001;
b = 1;
F1 = -(r^∧^2∗u(1))/(1+a∗u(1));
F2 = b∗(r^∧^2∗u(1))/(1+a∗u(1));
s = [F1;F2];
% –––––––––––––––––––––––––––––––
function u0 = pdex4ic(x)
u0 = [0;0];
% –––––––––––––––––––––––––––––––
function [pl,ql,pr,qr] = pdex4bc (xl,ul,xr,ur,t)
pl = [0;0];
ql = [1;1];
pr = [ur(1)-1; ur(2)];
qr = [0;0];

### C. Matlab Program for the Numerical Solution in (7)

Matlab program for finding the summation of the series in (12) is as follows:

r = 10;
a = 0.001;
x = linspace(0,1);
t = 100;
b = 1;
s0 = 0;
N = 100;
for n = 0 : 1:N+1;
s = s0+((4∗(-1)^∧^n∗cos(((2∗n+1)/2)∗pi∗x)∗
exp((((pi^∧^2∗ (2∗n+1)^∧^2)/4))∗t))/(pi∗ (2∗n+1)));
end
for n = 0:1:N+1;
g0 = 0;
g = g0+((-1)^∧^ (n)∗ (2∗n+1)∗pi∗(cos(((2∗n+1)∗pi∗x)
/2))∗(exp(((-r^∧^2/(1+a))-((2∗n+1)^∧^2∗pi^∧^2)/4)∗t))
/(((2∗n+1)^∧^2∗pi^∧^2+4∗(r^∧^2/(1+a)))/4));
end
u = (cosh(sqrt(r^∧^2/(1+a))∗x)/cosh(sqrt(r^∧^2/(1+a))));
v = b∗(1-s-u-g);
plot(x,v);

## Nomenclature and Units

[*S*]_em_: Substrate concentration in the enzyme membrane (mole/cm^3^)
[*P*]_em_: Product concentration in the enzyme membrane (mole/cm^3^)
[*S*]_aq_: Substrate concentration in the sample (mole/cm^3^)
[*P*]_aq_: Product concentration in the sample (mole/cm^3^)
*D*_*s*_: Diffusion coefficient of the substrate (mole/cm^3^)
*D*_*p*_: Diffusion coefficient of the product (mole/cm^3^)
*k*_3_: Rate constant for irreversible step (sec^−1^)
*K*_*M*_: Michaelis constant (mole/cm^3^)
[*E*]_tot_: Total concentration of free enzymes (mole/cm^3^)
*υ*: Number of product species (none)
*k*_*s*_ and *k*_*p*_: Distribution coefficient of the species between aqueous phase and enzyme membrane (none)
*k* = *k*_3_[*E*]_tot_/*K*_*M*_: Rate constant (sec^−1^)
*X* = *x*/*d*: Dimensionless distance (none)
*x*: Distance (cm)
*d*: Thickness of the enzyme membrane (cm)
*u*: Dimensionless concentration of substrate (none)
*v*: Dimensionless concentration of product (none)
*α* = *k*_*s*_[*S*]_aq_/*K*_*M*_: Parameter quantifying the degree of unsaturation/saturation of the catalytic kinetics (none)
*β* = *k*_*p*_[*P*]_aq_/*K*_*M*_: Parameter quantifying the degree of unsaturation/saturation of the catalytic kinetics (none)
$\gamma_{s}=\sqrt{kd^{2}/D_{s}}$: Diffusion rate constant of substrate through the polymer matrix reaction diffusion parameter (none)
*τ* = *Dt*/*d*^2^: Dimensionless time (none)
*t*: Time (sec)
*a* = *γ*_*s*_^2^/(1 + *α*): Dimensionless parameter (none)
*f*_*m*_ = (*π*^2^(2*m* + 1)^2^ + 4*a*)/4: Dimensionless parameter (none)
*f*_*n*_ = ((2*n* + 1)^2^*π*^2^/4) + *γ*_*s*_: Dimensionless parameter (none).
